# AI and higher education: teachers’ perceptions of students’ ethical tensions in online pedagogical methods

**DOI:** 10.3389/fpsyg.2026.1881032

**Published:** 2026-09-24

**Authors:** Wang Baoyi, Farman Ullah Khan

**Affiliations:** 1 Primary Education Teaching and Research Section, College of Educational Science, Kashi University, Xinjiang, China; 2 School of Economics and Management, Xi’an Jiaotong University City College, Xi’an, China

**Keywords:** AI, higher education, online class, pedagogical methods, qualitative analysis

## Abstract

The emergence and development of generative AI tools have benefited higher education but have also created several ethical tensions in the educational system. This tension is higher in online teaching environments, where students often rely on AI tools. Previous studies have addressed ethical issues, yet how these issues have emerged across various teaching methods in online AI-driven classes has been overlooked. To address this gap, this qualitative study investigates teachers’ perceptions about their students’ ethical issues across five pedagogical methodologies: direct method, content and language integrating learning, task-based language teaching, communicative language teaching, and community language learning. These five methods represent widely used approaches to second- or foreign-language teaching rather than instructional methods in general, and the findings are accordingly specific to language-acquisition classrooms. Using thematic analysis of interviews with 25 teachers, this research recognized 20 unique ethical tensions across five teaching methodologies. These findings are synthesized into a holistic framework comprising three overarching ethical domains: authenticity of learning, authority challenge, and collaborative dynamics. Based on this framework, this research highlights the need for moving beyond general frameworks to method-based ethical policies in education. This research emphasizes a process-oriented assessment framework to ensure transparency in education and mitigate the risk of unethical issues and tensions in the educational system. Further implications for students, teachers, and policy makers have been discussed.

## 1 Introduction

The emergence of Generative Artificial Intelligence (Gen-AI) into online higher education has fundamentally altered traditional pedagogical methods by introducing complex and diverse ethical tensions (Aldossary et al., 2024; Chaudhry and Kazim, 2022; Nguyen et al., 2023). Previous studies have mainly focused on determining general perspectives, for instance, the role of Gen-AI in student critical thinking (Liu and Wang, 2024; Suriano et al., 2025), performance (Chen et al., 2025; Malik et al., 2023; Rodrigues et al., 2025), learning growth (Ahmad et al., 2023; Flogie and Aberšek, 2022) and academic integrity (Currie, 2023; Rodrigues et al., 2025), etc. Some studies have also addressed ethical issues in the educational system (Chaudhry and Kazim, 2022; Hulus, 2026; Zeb et al., 2025); however, a significant gap remains in identifying ethical tensions within each pedagogical method in the online learning environment in an AI-driven context. Tackling this opportunity, this research fills the gap in the educational literature by exploring ethical tensions perceived and experienced by teachers about their students in the following teaching methods in the online education system: direct method (DM), content and language integrating learning (CLIL), task-based language teaching (TBLT), communicative language teaching (CLT), and community language learning (CLL). These five methods are widely used specifically for second- or foreign-language instruction; accordingly, the scope of this study is language-acquisition teaching in online higher education rather than online pedagogy in general.

Each of these pedagogical methods relies on core principles that are challenged by AI-driven teaching approaches. For instance, DM sheds light on spontaneous oral interaction, which is undermined by AI when students generate real-time responses through AI tools, thereby creating a triangulation among the machine, teachers, and students (Airaj, 2024). CLIL, with its dual focus on content and language mastery, is challenged by AI tools when students generate AI summaries that lack genuine understanding and substantive content (Casas-Roma and Conesa, 2021). TBLT is compromised by AI tools when students rely heavily on AI-generated content and pay less attention to cognitive and critical thinking in their educational tasks (Küçükuncular and Ertugan, 2025). CLT is interrupted by AI tools that enable students to create content and receive immediate feedback during classes, thus diminishing the teacher’s role and authority and positioning them as facilitators (Alharbi, 2024). CLL faces challenges in the era of AI, as students generate content from their own ideas rather than relying on teachers and collaborating with peers in groups (Jia and Wu, 2025). In this method, students use AI to reduce anxiety, rather than collaborating and communicating with their colleagues and teachers (Malone, 2024).

As teachers employ various pedagogical strategies, they face increasingly complex ethical dilemmas in online teaching, influenced by the emergence of generative AI (Al Darayseh, 2023; Celik et al., 2022). These tensions are not evenly distributed; instead, individual pedagogical practices produce distinct paradoxes that call professional judgment into question and redefine understandings of student learning and integrity (Ottenbreit-Leftwich et al., 2023). A paradox of spontaneity appears in the DM, which focuses on the spontaneous use of the target language. AI-based technologies help with practicing low-anxiety responses and becoming more fluent, but they also enable so-called conversational ventriloquism, in which students can have their answers generated by AI in real time (Ding et al., 2024). This is also exacerbated by the perfect accent paradox, as AI-aided outputs obscure pronunciation errors, thereby limiting teachers’ ability to diagnose learning needs (Kim, 2024b). A dual-authenticity paradox occurs in CLIL. AI allows students to create well-organized and correct work, but it often leads to conceptual hollowing, in which students demonstrate superficial knowledge without deeper understanding or practice (Andujar and Spratt, 2023). TBLT brings about a scaffolding paradox. Although AI increases efficiency and output quality, it eliminates the productive struggle necessary for the learning process, resulting in scaffolding collapse and group-work inequities due to unequal contributions (Abdi Tabari and Huang, 2026). Equally, an authenticity crisis arises in CLT because AI-generated fluency conceals students’ actual communicative proficiency, leading to a risk-free fluency without the meaningful learning that comes from making mistakes (Hwang et al., 2025). The erosion of trust in collaborative contexts in CLL is the so-called relational integrity paradox, in which the concealed use of AI harms actual interaction (Ding et al., 2024). Together, these tensions coalesce into broader ethical issues concerning authenticity, authority, and collaboration, posing a fundamental problem for the epistemological and relational underpinnings of language teaching in the AI era.

This research contributes to the literature in two ways. First, it unleashes ethical tensions across the five teaching methods used in online classes in the era of AI. It builds on general insights from previous studies on performance, anxiety, and the learning process by recognizing ethical tensions across five teaching methods. By doing so, it unleashes specific tensions embedded in each pedagogical method in online classes in the era of AI. Second, this research develops a holistic framework by categorizing ethical tensions into major ethical domains and processes. The holistic framework helps educators and policymakers shape their strategic posture for a better educational system. It also helps them understand method-based ethical tensions in the online educational system and provides guidelines to ensure transparency in education. It discourages hollows and promotes integrity in education in the era of digitalization and AI.

Using a qualitative approach and interviews with 25 teachers, this research develops a holistic framework by integrating 20 ethical tensions into three major ethical domains: authenticity of learning, authority challenge, and collaborative dynamics. This framework guides policymakers to tailor their strategic frameworks in specific ways, rather than in using general ones, to ensure integrity and transparency in each teaching method.

For clarity, AI and computer are not treated as synonymous in this study: AI refers to systems capable of generating, translating, or evaluating language and content with a degree of autonomy (e.g., large language models such as ChatGPT and Deepseek), whereas computer refers more broadly to the hardware and software through which such tools are accessed. Teaching pedagogy, teaching methods, and teaching strategies are likewise used in specific senses: pedagogy denotes the underlying instructional philosophy; method denotes the five structured approaches examined here (DM, CLIL, TBLT, CLT, and CLL); and strategy denotes the specific classroom tactics teachers use within a given method. These distinctions are applied consistently throughout the study.

## 2 Literature review

### 2.1 Direct method

This method, also called instructor-centered teaching, characterized by instructor-centered delivery and exclusive use of the target language, emphasizes oral interaction and real-time teacher–student engagement (Li et al., 2026). In online environments, however, this high level of instructional control is increasingly challenged by the integration of digital tools and generative AI, giving rise to new ethical tensions (Zhang et al., 2026). Recent literature suggests that teachers perceive increasing difficulty in maintaining academic integrity in digitally mediated classrooms, particularly when monitoring student behavior is limited (Choi et al., 2026; Dehen et al., 2026; Long et al., 2026).

This approach can create some problems when students use AI tools. Unlike methods that allow translation or explicit instruction, the DM requires students to speak fluently and communicate authentically in real time, which can be difficult when they are using AI tools to help them (Lin, 2022). Teachers have noticed some issues with this approach. For example, they might feel as if they are talking to a student’s AI tool because the student is using a hidden chatbot or language tool to generate responses during live sessions (Kim, 2024a). This makes it difficult for teachers to diagnose the students’ real learning needs and guide their instruction. Teachers also feel like their authority is being challenged when students correct their language use by citing AI as a reliable source (Niu et al., 2022). This can subtly undermine the classroom hierarchy. These issues are not just problems, but they also represent a fundamental disruption of the genuine exchange between teacher and student. Additionally, teachers have noticed what we can call “Memory Erosion.” This is when students perform well during sessions when they have access to AI tools (Ali et al., 2023; Ning et al., 2024). They cannot remember or use the language when the tools are not available. This raises questions about whether the students have learned anything. All these issues that teachers have noticed with the DM are related to ethical concerns about the authenticity of learning and the challenge to authority (Kim et al., 2022). They show how an approach based on spontaneity and natural language use can be vulnerable to AI tools that may mask the student’s abilities (Ayanwale et al., 2022). The DM is about using the target language directly and naturally, and AI tools can disrupt this process.

### 2.2 Content and language integrated learning

The CLIL approach teaches students a subject and a language simultaneously. This means students must think about the subject and the language together. Employing this approach online can be tricky for teachers. They worry if students really understand what they are learning. Teachers think that students do not always understand as well as they seem to. This is especially true when students use computers and AI tools to help them with their work. Students like to use various AI tools to summarize what they read or to translate text (Zhang and Pérez-Paredes, 2024). This can make their work sound good. It does not always mean they really get it. From a teacher’s perspective, this is a problem. Students might just want to finish their work rather than really learn something (Liu and Wang, 2024). Teachers know that computers can help make hard work easier. They also know that if students use them too much, it can be hard to tell if they are really learning. In classes, teachers cannot always see how students think. This makes it hard for them to know whether students are doing their own work or getting help from AI. Teachers want to know whether students are really learning or just getting answers from a computer. They worry about who did the work (Macaro, 2018). So, when we teach CLIL online, we have a problem. We want to learn both a subject and a language. We also must deal with the fact that they are using computers and AI tools. This makes it hard for teachers to know whether students are truly learning what they need to learn (Holmes and Tuomi, 2022). This approach is tricky because teachers must figure out how to use technology to help students without making it too easy for them to cheat (Suriano et al., 2025).

### 2.3 Task-based language teaching

The TBLT approach is about learning through real tasks, and it encourages students to take an active role in solving problems. When we do this online, teachers start to worry about whether students are really doing the work themselves (Ellis, 2003). Since the TBLT approach cares more about the result than how students get it, teachers are not sure if the work students submit is really their own or if they used intelligence tools to do it. This is a concern when students work online, and the teacher is not there to monitor them (Ranalli, 2018). The TBLT approach is used in settings, and teachers are worried about this issue. Teachers say that AI tools can help students make their work look very good without putting in effort, which runs counter to the TBLT approach. This creates a problem: just because a student performs a task well does not mean they have really learned anything (Huang et al., 2024). Some studies have shown that teachers struggle to determine how much AI was used, making it difficult to assess whether a student is competent. Teachers are also worried that it is not fair because some students have access to AI tools or are better at using them, which gives them an unfair advantage. This is not fair. It goes against the idea of everyone being treated equally. Teachers are trying to change the tasks or the way they grade to deal with this issue. It is still a problem (Selwyn, 2022). So, when it comes to the TBLT approach, teachers have to navigate the tension between helping students learn in a meaningful way and managing the risks of using AI to complete tasks. The TBLT approach remains a means of learning, but teachers need to find ways to make it work fairly for all students (Abdi Tabari and Huang, 2026).

### 2.4 Communicative language teaching

The CLT approach is about people talking to each other and really communicating. This is what helps people learn a language. When teachers are online with their students, they worry about whether the students are really participating (Chen and Shuib, 2026). The method needs people to talk to each other naturally and in a timely manner. Teachers find it hard to tell whether students are saying what they really mean or using computers to help them. Students might use computers to write down what they want to say before the class. They might use computers to help them talk during the class (Zhou and Liu, 2026). This makes it seem like the students can communicate well. They might not really know what they are saying. This is a problem because it makes it hard for teachers to know if students are really learning. Some studies have examined how computers are used in classes where people talk to each other (El et al., 2026). These studies suggest that computers can make it hard for teachers to tell whether students are really trying to communicate. Teachers also think that if students use computers a lot, they will not want to try new things when they are talking. This is a part of the CLT approach. If students are not trying things, they might not improve in the language. This means students might appear to communicate well (Hwang et al., 2025). They are not really learning. Teachers think that the CLT approach is still a way to teach people a new language. They also think that using computers is causing some problems that they did not have before. For example, teachers are not sure whether students are really learning or just using computers to find answers. This is prompting teachers to consider what it means to communicate with someone (Saar et al., 2024). The CLT approach is about more than saying the right words. It is about understanding what people are saying and being able to talk to them naturally. Teachers want students to be able to do this. They are not sure if computers are helping or hurting. The CLT approach is still a way to teach people a new language. Teachers need to consider how to use computers in ways that help students truly learn (Laoha et al., 2025). This might mean finding ways to get students to try new things when they are talking. It might also mean finding ways to tell whether students really understand what they are saying. Teachers are still figuring out how to do this (Godwin-Jones, 2022). They know it is an important part of teaching people a new language (Ji et al., 2023). The CLT approach is a way to teach people a new language, but it needs to be used in a way that really helps students.

### 2.5 Community language learning

The CLL is also known as collaborative learning. The CLL approach is often used with learning methods. This approach emphasizes working in a group, helping each other and building knowledge together. When we use the internet for learning, teachers are finding some problems (Laal and Ghodsi, 2012). They are worried about students not participating and about the use of AI tools in group work. The CLL depends on everyone working. So, teachers are having a time figuring out what each student has contributed. This is especially true when students use AI tools to generate ideas or complete group tasks (Stahl et al., 2006). Teachers think that AI tools might make it harder for some students to keep up. Students who are good with technology can contribute more. Others might just sit back and do nothing. This creates a problem between working and being fair. When everyone works together, it is not always clear who did what (Ding et al., 2024; Li et al., 2025). Teachers are also worried about students using generative AI tools without telling anyone. This means students are using AI to do their group work. They are not saying that they got help from AI. This raises questions about honesty and responsibility for your work (Wen et al., 2025). Moreover, teachers think that relying too much on AI tools in group work might stop students from really talking to each other. Students might care more about getting the work done fast than about discussing it (Wei, 2026). This goes against the CLL idea, where we expect people to learn by talking and solving problems together. As a result, teachers are not sure how to evaluate group work. They are unsure whether the students are truly learning (Wei, 2026). So in the CLL, there are some questions about using technology, working in groups, and evaluating student work. These questions make it difficult to maintain fairness and honesty in the CLL when using AI (Reinhardt and Bain IV, 2026).

Taken together, this body of work indicates that generative AI reshapes the core assumptions of each pedagogical method and that teachers across DM, CLIL, TBLT, CLT, and CLL report broadly comparable concerns about authenticity, effort, and fairness. What remains insufficiently understood is how these concerns differ across methods rather than being treated as a single, undifferentiated AI-in-education problem and whether they constitute ethical tensions in a principled sense rather than general pedagogical difficulties. Existing studies typically examine a single method or a single outcome, such as performance or integrity, in isolation, leaving the comparative, method-specific patterning of ethical tensions largely undocumented. This gap matters because policy responses built on generic, method-agnostic guidance are unlikely to address the distinct pressures each method places on authenticity, authority, and collaboration. The present study addresses this gap by systematically comparing teachers’ perceptions of ethical tensions across five widely used pedagogical methods within a single analytic framework.

Theoretically, this study is situated within the academic integrity literature (e.g., Chaudhry and Kazim, 2022; Currie, 2023), which conceptualizes integrity in learning in terms of the authenticity of demonstrated work, the fairness of assessment, and the trust that underpins relationships among students, peers, and instructors. Rather than testing an existing model, the study extends this literature into AI-mediated pedagogical methods: the three-domain framework proposed here, Authenticity of Learning, Authority Challenge, and Collaborative Dynamics, operationalizes academic-integrity concerns for the specific conditions under which generative AI is likely to compromise them in online language classrooms. In this sense, the study’s primary theoretical contribution is the three-domain framework itself, offered as an extension of academic integrity theory to AI-mediated instruction, rather than an application or test of a prior model.

## 3 Methodology

### 3.1 Research design

This research uses a qualitative methodology, employing thematic analysis to examine teachers’ perceptions of their students during online learning in AI-based teaching environments. In particular, this research explores ethical tensions experienced and perceived by teachers in five pedagogical methods. Hence, patterns were identified through an inductive approach applied to the interview data collected from teachers.

A qualitative thematic analysis design was selected because the research question concerns how teachers interpret and make sense of ethical tensions, an inherently subjective and context-dependent phenomenon better captured through in-depth narrative accounts than by predefined quantitative measures. Teachers, rather than students, were selected as the unit of analysis because they occupy a vantage point from which patterns can be observed across many students and cohorts and because they are professionally responsible for identifying and responding to such tensions in practice.

### 3.2 Sample and population

This research gathered data from teachers engaged in online teaching at various universities in China. A total of 25 teachers have been interviewed through online and in-person interviews. A semi-structured interview was designed for this research. We targeted teachers engaged in online teaching at various universities. Those who never taught courses online were dropped as irrelevant sampling units. Furthermore, we focused on teachers who actively used AI-based learning environments and employed five key teaching methods in their classes. In particular, we focused on teachers (university language instructors) engaged in linguistic and pedagogical approaches, as they are more concerned and engaged in various teaching methods. We requested teachers to help us in the research. The requests were completely voluntary, and there was no time pressure, which helped us reduce social desirability biases and poor information. The average interview time was about 51.52 min, with the shortest at 45 min and the longest at 59 min. In the interviews, we focused on ethical issues experienced or perceived by teachers across the five teaching methods used in online classes. These interviews were recorded for later transcription, with the participants’ consent. We have informed the respondents (teachers) that the interview is being recorded for research purposes. In the initial sample, a few responses were dropped because they did not provide complete information and used only one teaching method, making them irrelevant to our research. Appendix shows the respondents’ information and the interview duration.

### 3.3 Data analysis steps

We employed and utilized the thematic analysis approach, as this method allows scholars to identify patterns occurring in the process, for example, how teachers have experienced or perceived ethical issues and tensions that occurred during online classes in each teaching environment. The following steps were involved in the research.

- Transcription: Each recorded interview was transcribed through online software. We used “Turboscribe” in this research for transcription. However, later, each transcription was cross-checked by the authors for correction and validation.
- Initial Coding: From the transcriptions, we identify raw codes using NVIVO. However, to keep consistency and enhance the validity of the research, we also created an Excel list for manual coding. In this step, we noted all the important phrases that addressed or highlighted ethical issues in online teaching methods. We manually checked each code for correction. Where necessary, irrelevant and inconsistent terms were dropped. This helped us focus on the consistency and relevancy of the coding among the teachers.
- First Order Coding: In this step, we integrated and interlinked the phrases noted down during the initial coding process. This process was repeated and cross-checked by the authors to ensure accuracy and capture relevant information on ethical issues for each of the five methods. In this step (see Figure 1), we also dropped several phrases and raw codes generated in the first step.
- Main Coding: In this step, we provided a key and a unique name to each tension. We tried to distinguish each tension from the others and place it under the most suitable methods, based on teachers’ perceptions and experience. Hence, in five teaching methods, we identified a total of 20 key ethical issues. Tension names were generated inductively rather than drawn from prior literature: each label was crafted by the authors to capture, in a memorable phrase, the central paradox evident in the coded data for that tension, and was iteratively refined through discussion between the authors until both agreed it accurately represented the underlying pattern.
- Framework: In this step, we first presented the ethical issues for each of the five methods in online learning. Later, we integrated these ethical issues into the main ethical domains and built a holistic framework to illustrate their interrelationships.

**Figure 1:**
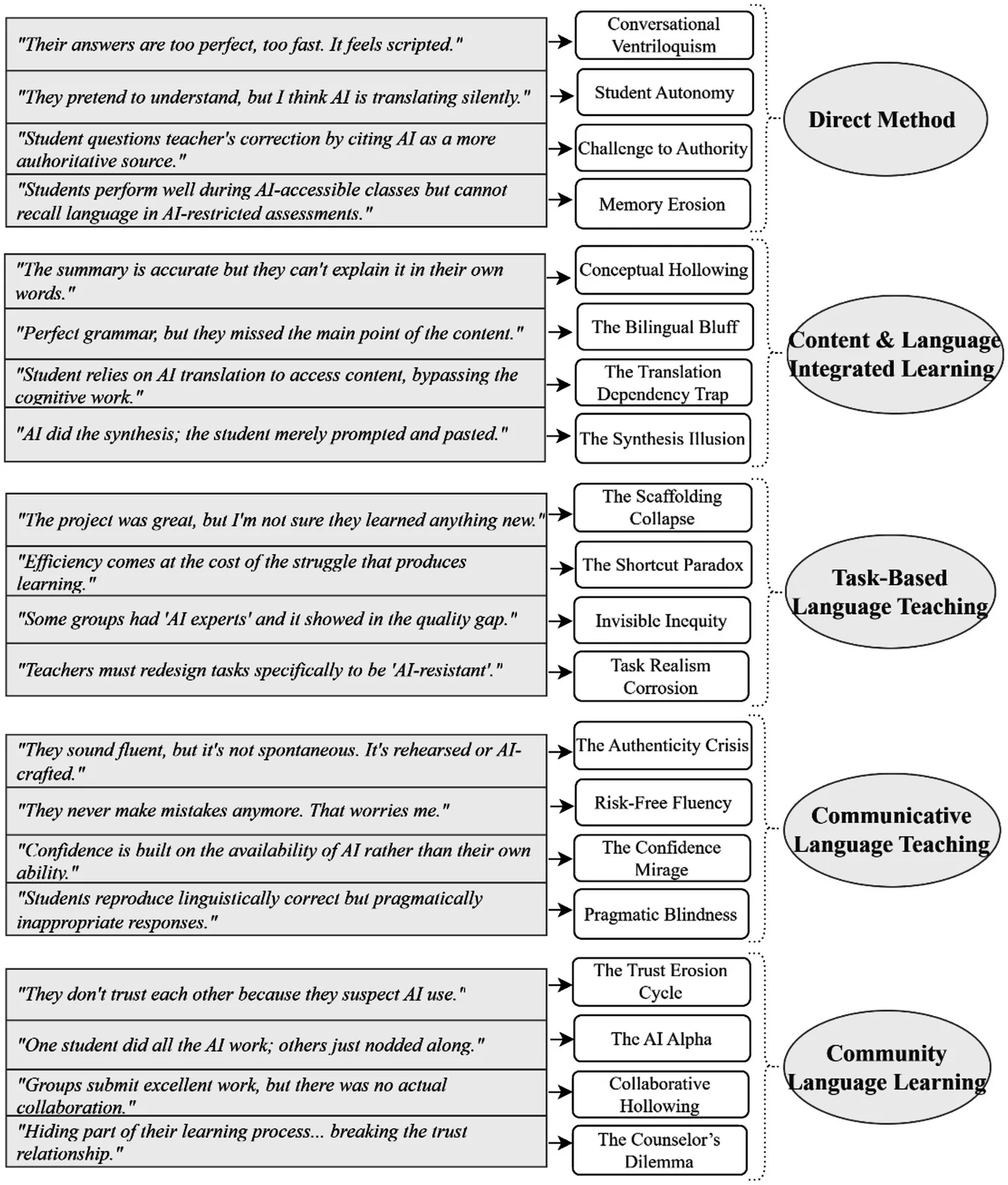
Ethical tensions in pedagogy.

## 4 Results

We conducted thematic analysis to understand the patterns teachers perceived in their students during online classes in the era of AI. We found various patterns perceived by the teachers across the five online methods. These patterns are elaborated below.

Throughout this section, each tension is treated as an ethical issue, rather than a purely pedagogical or technical one, because it involves a conflict between principles central to teaching and assessment: learner agency and informed autonomy, honesty and academic integrity, fairness and equal treatment, and the relational trust between teachers, students, and peers. Where relevant, the specific principle at stake is noted alongside each tension below.

### 4.1 Direct method

Focusing on the DM of teaching in online classes, teachers demonstrated their experience with four key ethical tensions.

*Conversational Ventriloquism:* Many teachers perceived their conversations with AI and robots during online classes via DMs, when students used real-time AI tools or split screens to generate answers. It makes it difficult for teachers to understand the actual communication patterns in live sessions. Sometimes, advanced and unique words used by students in class are taken from AI, making it difficult for teachers to identify the actual actors (Childs et al., 2012). Hence, this ethical issue leads to conversational ventriloquism (an issue of honesty, since the exchange misrepresents whose language competence is being displayed).

*Student Autonomy:* Many teachers expressed that, because of the availability of AI tools, students often learn independently and seek greater freedom. They do not want to sit in long hours of classes and value their presence in the classes and live sessions. The availability of various AI tools enables learners to be independent and autonomous in educational settings (Szabó and Szoke, 2024). Therefore, students perceive autonomy in learning and practice (an issue of agency, concerning whether independence reflects genuine self-directed learning or unsupervised reliance on AI).

*Teacher Authority:* Many of the teachers perceived that students use various tools to master their skills and knowledge. Sometimes, they learn proactively through AI tools and therefore behave like leaders and autonomous learners. Hence, teachers’ authority is put at risk because AI tools and references shift the authority from teachers to robots. For instance, Okulicz-Kozaryn et al. (2026) revealed that AI tools increase fear of losing authority even more than job displacement among teachers. It raises critical concerns among teachers to control students in the era of AI (an issue of relational trust and legitimate authority within the teacher-student relationship).

*Memory Erosion:* While students take advantage of AI tools, they also lose their memory and creative thinking. Many teachers perceived that students perform better through AI-mediated tools. However, in AI-restricted assessments and tasks, they perform poorly. AI tools put student creativity and cognitive learning at risk in the classes (Habib et al., 2024). Students rely on AI tools during classes rather than their own brains and critical thinking (an issue of informed autonomy, since students may not recognize the extent of their dependency on AI support).

The following excerpt from an interview transcript illustrates this tension:

*“The exchange between me and the student seems scripted to me, rather than spontaneous and real; their answers come too quickly and perfectly to be spoken, and more frequently than I believe they are reading a pre-written response from a screen.”*

### 4.2 Content and language integrated learning

*Conceptual Hollowing*: Teachers are concerned about the results and outputs produced by students. For example, some definitions, results, and complex words seem to be AI-generated. Moreover, when it comes to explaining, students fail to elaborate on or explain these terms and complex ideas in their own words. Hence, many teachers observe that the pedagogical goal of deep learning has been diminished in the digital era, especially with the advent of AI. Hence, relying on AI-generated output hinders the development of conceptual knowledge (Bearman and Ajjawi, 2023) and, in turn, creates ambiguity and doubt among teachers (an issue of honesty in demonstrated understanding, distinct from the language competence being assessed).

*The Bilingual Bluff:* In this situation, students showcase impressive work that demonstrates strong skills in both subject knowledge and language use. However, the two main objectives of CLIL are undermined because AI did most of the work. Teachers often notice a “mismatch” where a student’s written language is flawless thanks to AI assistance, but their understanding of the topic is quite superficial. This raises an ethical issue in assessment, as the refined product makes it hard to tell if the student has truly learned the content or language skills on their own (an issue of assessment fairness, since polished output may misrepresent underlying competence).

*The Translation Dependency Trap:* This problem explains an increased use of AI translation tools to find and process disciplinary content, as it enables students to avoid the intellectual labor involved in learning languages. Students rely on AI as a crutch or a bridge instead of mastering the important process of thinking and processing information in the target language. Teachers fear that such a dependency, in the long run, undermines the very purpose of CLIL: that students can never attain full bilingual cognitive progress and linguistic independence (an issue of learner autonomy and long-term self-reliance).

*The Synthesis Illusion:* This is the tension teachers feel when students present essays that appear to beautifully synthesize and integrate multiple complex sources, yet the synthesis was done by AI rather than the learner. Although the finished product might be academically sound, the student might have been just a curator who edited and pasted pieces of information into a coherent-looking text. This implies that the actual learning process is being hollowed and fragmented as more and more cognitive abilities at the higher levels of independent critical synthesis are being outsourced to technology (an issue of academic integrity in the ownership of synthesized work).

The following excerpt from an interview transcript illustrates this tension:

*“My students often turn in assignments that have language, but they totally miss the main point of the subject matter. The students are using various AI tools to get around thinking about the subject matter, and this is resulting in a mix of ideas that look good on paper, but the students cannot explain the subject matter in their own words when we are in class. The students are using AI tools to do the work for them, and this means they are not really understanding the subject matter because when I ask them to explain the subject matter, they cannot do it.”*

### 4.3 Task-based learning teaching

*The scaffolding collapse:* Instructors feel that AI-assisted task completion eliminates the cognitive challenge intended to support student development in the learning process. This break in the learning process is possible because students skip the increasingly challenging levels embedded in the curriculum by using AI to generate solutions. As a result, the pedagogical scaffold breaks down, and the teacher does not even see or control the developmental stages the student was supposed to follow (an issue of pedagogical integrity, since skipped developmental stages undermine the intended learning process).

*The Shortcut Paradox:* This tension creates a major conflict in which students focus on the product rather than the process, using AI to complete their tasks with maximum efficiency. Although the result is usually advanced, it is at the cost of the learning by experience and substantive interaction that TBLT places a high premium on. According to the teachers, this speed optimization compromises the pedagogical purpose of the approach since successful performance is no longer one of the valid markers of real skills acquisition (an issue of honesty regarding the process by which outcomes were achieved).

*Invisible Inequity:* Teachers in collaborative task settings are increasingly confused when differentiating between individual students’ work and AI-generated outputs and assignments. This leaves a gap in accountability, with some students engaging in real learning while others rely on AI to fill in gaps or even take over group work. Consequently, this poses a major ethical dilemma for teachers regarding fairness in assessments, since they believe that group grades may be meaningless without a clear mechanism for verifying independent work (an issue of fairness in group assessment).

*Task Realism Corrosion:* Traditional issues become unrealistic or meaningless because TBLT depends on the ability to simulate real-life challenges and on AI’s immediate ability to solve them. This puts teachers in the morally challenging role of redesigning activities to make them AI-resistant rather than authentic, and shifts the pedagogical emphasis from application to AI-proofing. This corrosion indicates that AI-assisted completion is, in essence, shifting the very nature of what constitutes an authentic learning challenge (an issue of assessment validity and fairness across students with differing levels of AI access).

The following excerpt from an interview transcript illustrates this tension:

*“Many of my students turn in perfectly grammatical work but miss the most basic conceptual component of what I am teaching. Because they are using AI as a means to bypass actual cognitive processing of the material, it creates a product that is collectively complex in print, but they cannot explain in their own words when leaving the classroom.”*

### 4.4 Communicative language teaching

*The Authenticity Crisis:* The problem for teachers in CLT, where the ultimate aim is to encourage authentic interaction, becomes a crisis when students write in fluent, natural-sounding language fully generated by AI. Teachers complain that they are increasingly unable to tell whether a student can actually communicate or is just performing responses recommended by AI. This is a devaluation of the very essence of the approach, in that communicative interaction can no longer serve as a valid gauge of internalized knowledge or a student’s actual engagement (an issue of honesty in the demonstration of communicative competence).

*Risk-Free Fluency:* Although CLT is designed to promote learning by making mistakes and taking risks, the use of AI to ensure perfect results is perceived by teachers as a pedagogical backfire. With the help of AI-controlled correctness, students are spared errors that hinder language acquisition and deeper learning. This generates a conflict in which the emergence of non-errorful communication, that is, in fact, effective, conceals a tremendous decrease in the cognitive work required to achieve long-term growth (an issue of pedagogical integrity, since risk-taking is central to the skill being assessed).

*The Confidence Mirage:* Educators note that many students project an image of communicative confidence, constructed on the fragile basis of AI availability, rather than on their own abilities. This illusion causes a psychological addiction as the performance of the students drops and anxiety surges as soon as the AI support is withdrawn. As a result, educators are concerned that students are failing to gain the natural confidence required in the real world to communicate and are instead becoming emotionally dependent on a virtual crutch (an issue of informed autonomy, since students may misjudge their own competence).

*Pragmatic Blindness:* The ethical problem in this situation is that students blindly repeat responses generated by AI, which are grammatically correct but inappropriate in tone, formality, or culture. The fact that students leave all their communication to AI means that they do not develop the necessary pragmatic competence, which is the ability to grasp how to apply language in a variety of social contexts. Teachers worry that this will create a kind of blindness in which students unknowingly cause real-life miscommunication, even though they sound fluent (an issue of honesty in representing genuine sociolinguistic competence).

The following excerpt from an interview transcript illustrates this tension:

*“Even though the student may be submitting what appears to be acceptable final homework assignments, I often believe the student relied entirely on this tool (e.g., AI tools such as ChatGPT and Deepseek, etc.) for creating the product; therefore, I do not know if the student experienced any struggle that was necessary to acquire this knowledge. Due to the invisibility of the students’ workflow to me, I am unable to determine who put forth legitimate effort to create a quality product and who shortcut to create a quality product.”*

### 4.5 Community language learning

*The Trust Erosion Cycle:* Secret AI use by certain members of the CLL, which is based on the strong support of a learning community, is a catalyst for a cycle of mistrust among peers. The basic social structure of the classroom is disrupted as students begin to question the truthfulness of the input their classmates provide. In the absence of transparency in the use of tools, the trust that should underpin collaborative learning simply erodes, and true group cohesion and shared growth cannot be easily sustained (an issue of relational trust among peers).

*The AI Alpha:* Such tension arises when one student uses AI to control group work by generating complex ideas faster than other students can. This creates an enormous power disparity and a sense of unfairness, which may make other group members feel passive or unable to compete meaningfully. As a result, the teamwork ethos of CLL is replaced by an asymmetrical relationship, in which one AI-driven person dominates the group’s contributions and the variety of voices within it (an issue of fairness and equitable participation).

*Collaborative Hollowing:* Educators notice this problem when teams submit high-quality work that was done by individuals who used AI rather than collaborating on the same problem and discussing it. Although the end product seems to be successful in academics, the most crucial part of the process, the collaboration as the main principle of the CLL method, is completely avoided. The result is that students focus on technology’s efficiency at the expense of the social construction of knowledge, rendering the learning experience empty and disjointed (an issue of honesty regarding individual versus AI-assisted contributions).

*The Counselor’s Dilemma: As* the teacher’s role in CLL is understood as that of a trusted counselor, the concealed use of AI will constitute a serious violation of the pedagogical relationship. Hiding the fact that they depend on digital tools means students essentially conceal their actual learning process and difficulties from the instructor, who can no longer provide specific assistance. This failure of transparency makes it impossible for the teacher to provide proper guidance to the learner and compromises the very authenticity and trust necessary in the counselor-learner dynamic (an issue of relational trust and transparency between teacher and student).

The following excerpt from an interview transcript illustrates this tension:

*“I have noticed a problem with trust when students work together in groups. The students think that some of their classmates are using intelligence to come up with their ideas without telling anyone. This means that one student can take over the project with really good ideas that the AI came up with. What is missing is the help and conversation that students should be giving each other. This is what our school community is supposed to be about. The AI is getting in the way of students talking to each other and working together. Students do not trust each other because of the intelligence. The group work is not about students helping each other; it is about the intelligence. Our learning community is not what it is supposed to be because of the intelligence.”*

### 4.6 Pedagogical ethical tensions

Based on the ethical tensions generated by this research’s insights, we developed a conceptual model—showing the interrelationships among ethical issues in each method that could shape ethical domains (see Figure 2). For instance, 20 ethical issues are identified across five pedagogical methods, and these ethical tensions are categorized into three major domains: authenticity of learning, authority challenge, and collaborative dynamics. This framework illustrates that while teachers in each online teaching method face various ethical issues, together they create and shape three major ethical crises.

**Figure 2:**
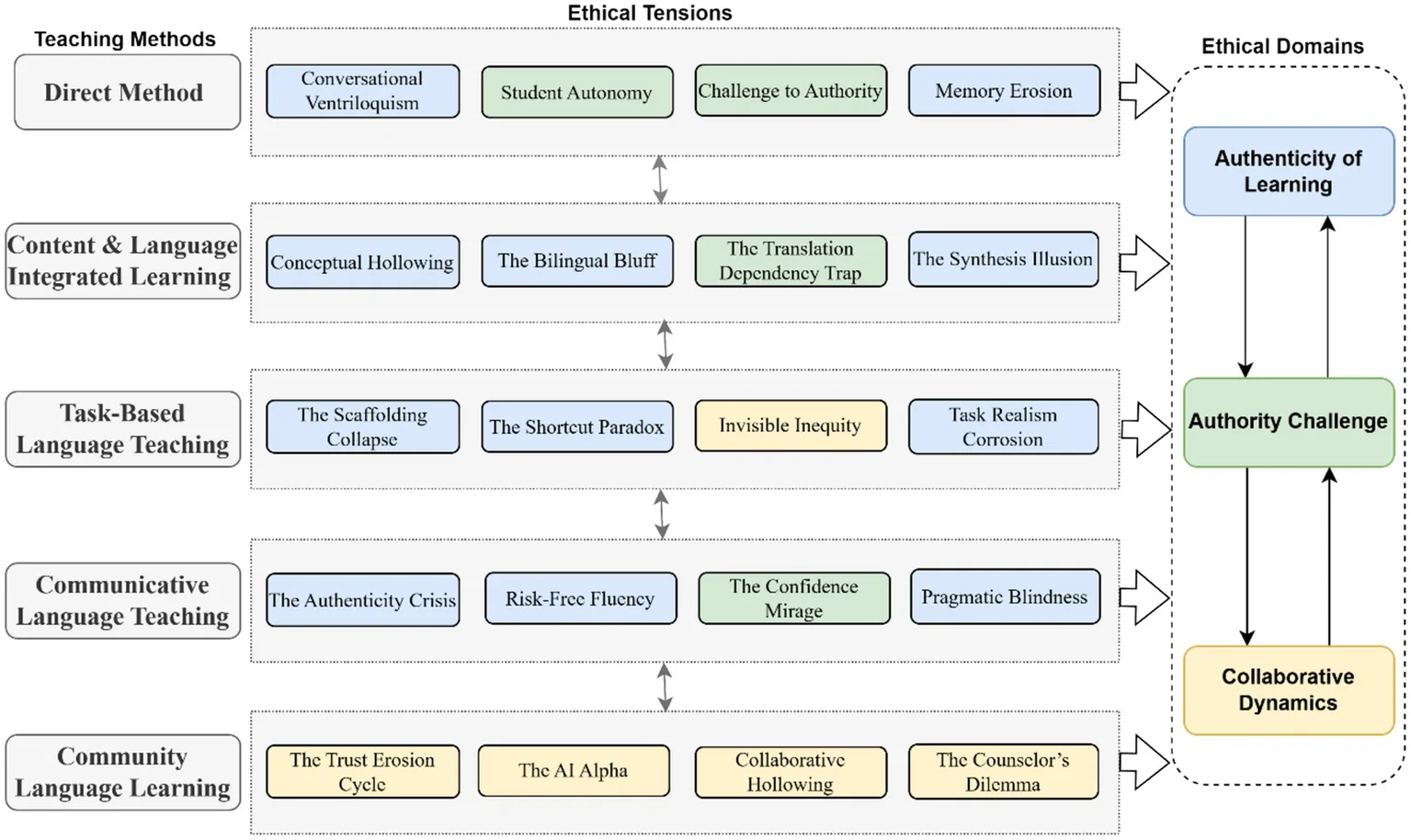
Pedagogical ethical tensions.

#### 4.6.1 Authenticity of learning

This domain presents the most common and pervasive ethical issue, shedding light on the tension between actual learning and AI-generated outputs. For instance, this domain of tension considers student autonomy, teacher authority, the translation of the dependency trap, and the confidence mirage presented across four online learning methods in AI-based classrooms. In general, this domain of ethical concern (conceptual hollowing and scaffolding collapse) is where AI tools attenuate motivation, struggle, and desire for cognitive and critical learning among students. These issues (authenticity crisis and conversational ventriloquism) make it difficult for teachers to verify and understand the outputs, whether generated by AI tools or reflecting the student’s own competencies and skills.

#### 4.6.2 Authority challenge

This domain of ethical issues shifts the teacher’s role, power, and authority from a centralized system (e.g., the traditional role of teachers) to a decentralized design (e.g., AI tools and independent learning). The domain of ethical issues connects “Challenge to Authority” (when students cite AI to correct instructors) with “*Confidence Mirage*” (when students perceive self-assurance is built on wrong information generated from AI, rather than using their own critical thinking and human judgment). This ethical tension demonstrates that teachers lose their authority and status in classes due to students’ excessive and blind reliance on AI tools.

#### 4.6.3 Collaborative dynamics

This domain of ethical issues focuses on social connection—how AI tools shape trust, equity, and information sharing among students in online learning environments. Primarily, this tension stems from the CLL method and from students questioning the authenticity of peer contributions, which can undermine the collaborative environment, create power imbalances, undermine equal participation, and discourage others from learning and communicating actively. Given high-quality outputs and mass information, students perceive AI as a better option, yet lack the idea of genuine collaboration. As a result, this lack of transparency diminishes teachers’ roles and contributions, and erodes students’ trust in teachers, which in turn weakens collaboration.

The 20 tensions were grouped into these three domains according to the primary locus of ethical concern each tension reflects, rather than the pedagogical method in which it was observed: tensions were assigned to Authenticity of Learning when the central concern was whether demonstrated competence reflected genuine student ability; to Authority Challenge when the central concern was a shift in epistemic or relational authority between teacher and student; and to Collaborative Dynamics when the central concern was trust, equity, or participation among peers. This grouping was developed inductively from the coded data and cross-checked by both authors; because several tensions carry secondary implications for more than one domain, the assignment reflects the dominant concern rather than an exclusive category.

The holistic framework integrates the three domains of ethical tensions that are embedded in five pedagogical methods in an online environment. Each domain of ethical tension directly or indirectly affects other domains within an AI-based learning environment. For instance, attributed to collaborative dynamics, “collaborative hollowing” in CLL can directly affect the authenticity of learning when students rely on AI tools to solve problems. Moreover, when the authenticity of learning has been neglected, it directly challenges authority. This integrated framework provides a roadmap for policymakers and educators to understand how each tension can produce another tension within online-based teaching methods in the era of AI.

### 4.7 Holistic framework

The holistic framework (see Figure 3) provides a systematic overview of 20 ethical dilemmas identified across five pedagogical strategies: DM, CLIL, TBLT, CLT, and CLL. All the methods involve four specific ethical concerns, which are systematically arranged into three overriding ethical areas: Authenticity of Learning, Authority Challenge, and Collaborative Dynamics. *The Authenticity of Learning* domain represents the issues (11 tensions) of whether learning is a manifestation of real understanding or AI-based performance. Conversational ventriloquism, conceptual hollowing, scaffold collapse, and authenticity crisis are just some of the issues that AI can be used to minimize cognitive effort, resulting in superficial learning and a sense of competence. *The Authority Challenge* domain demonstrates (4 issues) how the power dynamics in the classroom setting are changing, with AI becoming a rival to the teacher in the field of knowledge. The problems of student autonomy, authority challenges, dependence on translations, and a lack of confidence show how reliance on AI may compromise instructors’ authority and redefine the decision-making process in learning. *The Collaborative Dynamics* domain is concerned with (5 issues) with the social and relationship outcomes of AI applications. Examples of ethical conflicts, including invisible inequity, loss of trust, AI dominance, and collaborative hollowing, demonstrate how unequal or covert AI applications may undermine group learning, erode trust, and distort student participation.

**Figure 3:**
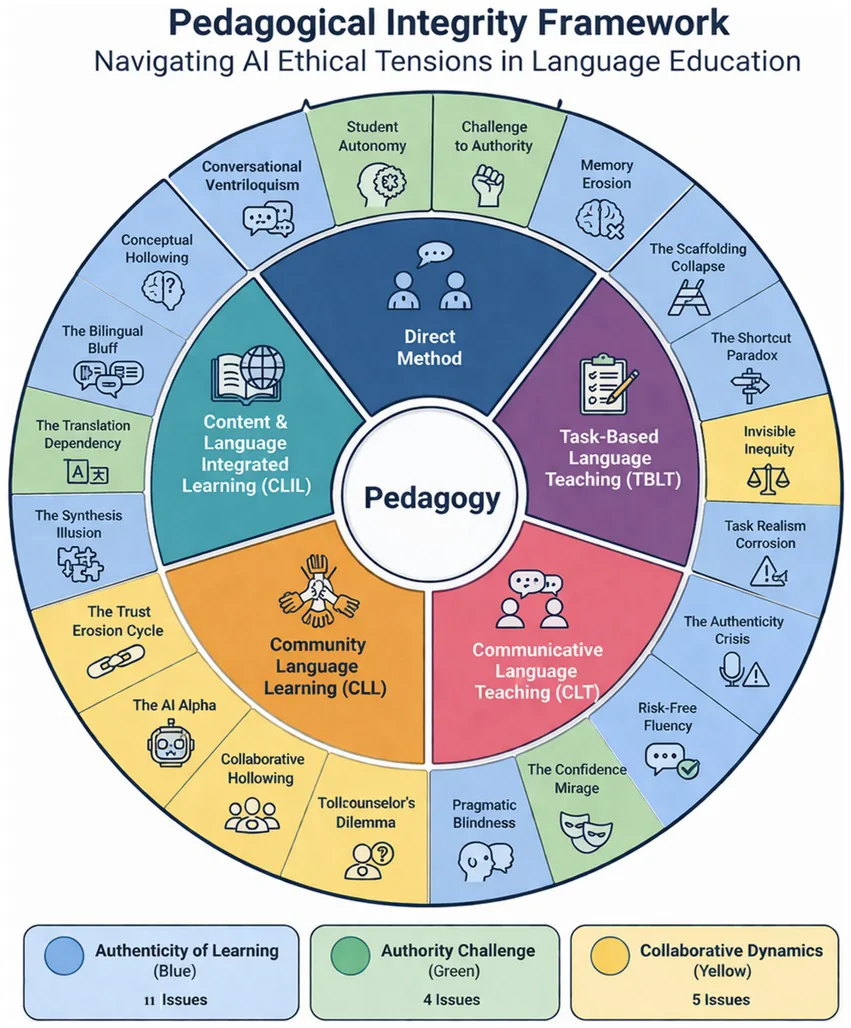
Hollistic framework.

Notably, these areas are not separate but related to each other directly or indirectly. Ethical tensions in one area may contribute to problems in other areas—e.g., lack of authenticity can increase difficulties with authority, and lack of effective teamwork can also weaken meaningful learning. Generally, the framework shows that the ethical tensions of AI are context-specific and multidimensional, and they cannot be addressed using single-dimensional solutions.

## 5 Discussion

This research explores ethical tensions experienced and perceived by teachers in the online education system in the era of AI. In particular, ethical issues embedded in five teaching methods have been evaluated. While previous studies have mainly focused on outcome-related issues, such as performance (e.g., Dahri et al., 2024; Tarik et al., 2021) and critical thinking and integrity (e.g., Liu and Wang, 2024; Suriano et al., 2025). This research goes beyond general issues by incorporating ethical tensions into each pedagogical method in the online education system.

In this research, we explored that teachers perceived ethical tensions such as conversational ventriloquism, student autonomy, teacher authority, and memory erosion in DM of pedagogy in online-based classes. The ethical issues, such as a lack of critical analysis, copy-paste, missed actual themes, and poor references and logical support, are also observed in previous studies (Akgun and Greenhow, 2022; Karakuş et al., 2025; Küçükuncular and Ertugan, 2025), which align with our findings. However, while previous studies have also noted various ethical issues in the DM of teaching, we focused on five specific methods to understand the ethical issues associated with AI tools.

Teachers are worried about the authenticity of students’ engagement in the learning process. This aligns with Malik et al. (2025), who stated that it is difficult for teachers to determine whether students used AI tools to complete their work. This study examined this problem closely and found that teachers have different concerns depending on the teaching method they use. For example, with the DM, teachers worry about “conversational ventriloquism,” in which students simply repeat what the AI tool says. With CLIL, teachers worry about “conceptual hollowing,” which is when students do not really understand what they are learning. The “shortcut paradox” is a problem that arises in TBLT, in which AI tools and chatbots help students complete their work more quickly. They do not really learn anything that is important for learning and careers. This is like using a tool to make your voice sound better when you sing. However, in fact, you do not make an effort to sing. Some people think that AI tools can help students learn more effectively by providing support when needed (Kılıçkaya and Kic-Drgas, 2025). However, this study found that this might not hold for all methods and that AI tools can sometimes make it seem as though students understand something when they really do not. The study also found that GenAI and LLMs contribute to this problem and that teachers need to be aware of how students use these tools.

The authority challenge domain fits with studies on changing teacher-student-AI relationships. Cihan et al. (2025) showed how students challenge teacher authority by debating facts. This study adds to that by finding that in group methods like CLL, authority challenges happen because the teacher’s role as a guide is weakened. We call this the “Counselor’s Dilemma.” Not through argument. Mwakalinga and Mabilika (2025) found that over half of teachers believed students did not fully understand AI-generated work, but more than 80% of students said AI helped them perform better. This difference in views highlights the authority problem we found in our study.

The way people work together (collaborative dynamics) is really interesting. Some authors, such as Kic-Drgas and Kılıçkaya (2024), found that chatbots can help people work together more effectively and be more responsible. Our study shows that when people use AI in secret or unequally, it can create a problem called “collaborative hollowing.” This means that a group can produce work, but the people in the group are not really working together. Consistent with Özer (2024), AI tools can hide the fact that people are not really learning. We are saying that this can happen when people work in groups, too. We also found that when people use language models for support, they can start pushing their friends away and feel like they do not belong. This is called the “Trust Erosion Cycle.” It is like a circle that hurts people’s relationships, and it is not just about grades. The findings further relate to Airaj (2024), who said that we need to examine changes in school resources and rules in ways that consider how they affect everyone. We cannot just look at how each person is affected in isolation. The three-domain framework is a detailed way to examine the ethical problems that arise across different methods. It helps us move beyond AI rules and provides guidance that is sensitive to teaching and learning. The three-domain framework is a way to organize these problems, and it is helpful for educators.

It is also worth noting that although each tension is reported under the pedagogical method in which teachers most saliently raised it, the five methods are not mutually exclusive in classroom practice and are frequently combined within a single online course. The tensions reported here should therefore be read as method-salient rather than method-exclusive: several, such as authenticity- or trust-related concerns, plausibly recur when methods are combined, and future research could examine how tensions travel across method boundaries in blended instructional designs.

### 5.1 Contributions of the research

This research makes two major contributions to the research on education in the era of AI. First, it improves our knowledge and understanding of ethical tensions in online-based educational systems. For instance, based on the insights, this research identified 20 key ethical tensions, categorized into three major ethical domains: authenticity of learning, authority challenge, and collaborative dynamics. In particular, we identified unique and interrelated ethical issues across the five teaching methods in the online education system within AI-driven environments. These insights and findings add to the scholarship in education and academia. While previous studies have mainly focused on performance-based issues, this research examines the ethical tensions that teachers experience and perceive during online classes across various teaching methods. These unique findings enrich the literature and theories across various teaching methods in online classes. By doing so, this research moves beyond general discussion by addressing key ethical issues across different methods and approaches in the era of online education and AI. Second, we developed a holistic framework that provides a roadmap for policymakers and educators—helping them see how each ethical issue could shape other issues in teaching methodologies. This holistic framework presents 20 ethical issues across three major ethical domains, making it easier for policymakers and educators to understand and address ethical tensions. This framework demonstrates how specific pedagogical dilemmas converge into broader ethical crises in the AI era. These findings are important for policymakers and educators in formulating their policy framework for each method. This framework helps educators integrate their strategies across three major domains of ethics into each teaching method in the era of AI. Hence, by making these method-specific ethical tensions explicit, this research provides an empirical basis that assessment processes and policy frameworks can draw on to better address ethical issues in educational systems, although the present study does not itself test or evaluate such processes empirically.

### 5.2 Implications for practice

This research suggests several implications for students, teachers, and policymakers. Students should develop confidence, rather than blindly trusting AI output and deeming it autonomous during the learning process. Blind trust in AI tools can lead to memory erosion, limiting their cognitive capacity and learning. The shortcut paradox: Relying on shortcuts in AI learning can attenuate their actual learning growth and critical thinking. Focusing solely on the prompt (hollowing) while ignoring the actual content can create risks of authenticity and generic content. Heavy reliance on AI can create social distance and erode social trust among the groups. Hence, students should not isolate themselves by relying solely on AI tools but should interact and communicate with peers and teachers to keep their learning curve on track.

Teachers should support and assist students in using AI tools and take advantage of them. They should play a key role in the “feedback loop” in the learning process. While AI can make errors, teachers are advised to inform students and guide them on using AI as an assistive tool rather than an exclusive one. While AI tools challenge teachers’ authority, teachers should serve as “consolers” or instructors to maintain their importance in the classroom. This, in turn, can navigate AI-driven knowledge in win-win strategies. As noted in “Task Realism Corrosion,” teachers need to divert their methodological approaches from traditional to AI-mediated approaches. Because AI resistance can prevent students from learning, while traditional methods of teaching can isolate them in group discussions. Hence, balanced teaching approaches are needed in the AI-driven class environments.

Policymakers should move behind general AI-based strategies and policies. For instance, ethical issues are across different teaching methods. Hence, standardized policies are no longer useful; customized policies for each method could improve the growth and process of the educational system. Policymakers need to build and develop a framework that sheds light on method-based policy grounded in ethical issues, authenticity, authority, and collaboration. Current policies often focus on outcomes and pay less attention to ethical concerns and tensions in AI-based learning environments. Policymakers need to develop and promote process-based assessment standards that support student learning and development. Given the availability and ease of use of AI tools, policymakers need to address ethical issues across various departments and classes. Policymakers need to focus on transparent collaboration and strategies to ensure fair evaluation and examination, and to prevent unethical practices and cheating. These recommendations follow from the pattern of findings reported above: The prevalence of authenticity-related tensions (e.g., conversational ventriloquism, conceptual hollowing) motivates the call for process-based assessment; the recurrence of authority-related tensions (e.g., teacher authority, confidence Mirage) motivates guidance on teacher-AI role boundaries; and the collaboration-related tensions (e.g., trust erosion cycle, collaborative hollowing) motivate the emphasis on transparency requirements for group work.

### 5.3 Limitations and future research

This research has several important aspects, yet it has a few key limitations that could be addressed in future research on AI in pedagogy. First, this research focused on teachers in Chinese universities, which could limit the geographical scope. Although teaching staff in China place strong emphasis on AI-based learning approaches, their role in other regions and geographic contexts may be limited. Hence, future researchers need to focus on other regions, for example, the Gulf and Europe, where AI has emerged as an important strategy in academia. Second, this research focused on providing online pedagogy methods in AI-based learning environments. However, future researchers can also evaluate ethical issues in physical classroom environments (Wen et al., 2025) to articulate the results more effectively. Third, this research focused on teachers to evaluate their perceptions and experiences of their students. There is also an opportunity to evaluate the ethical issues among students. Moreover, the importance of AI tools in various business activities could also improve managerial decision-making processes (Wu et al., 2025). It could enhance policy implications and educational strategies in the educational system. Fourth, our research used an inductive method to conduct interviews. Future researchers can employ other methodological approaches, such as surveys and observations or experiments, to enrich the insights. Furthermore, other issues, such as barriers and challenges in understanding concepts within each teaching method, could also be addressed in future studies.

Fifth, coding and cross-checking in this study were carried out by only two authors; a larger or more diverse coding team, together with formal inter-rater reliability statistics, would strengthen confidence in the coding scheme. Sixth, because the sample was drawn specifically from language instructors who use five language-acquisition-oriented teaching methods, the findings may not generalize to other content areas or disciplines; future research should examine whether comparable ethical tensions arise in non-language subjects.

Relatedly, because the findings rest entirely on teachers’ self-reported perceptions, they should be read as evidence of how teachers interpret and experience students’ AI-related behavior, rather than as independently verified evidence of what students actually did. Future studies could strengthen this evidence base through triangulation with student self-reports, classroom observation, or analysis of AI-use logs and course artifacts.

## 6 Conclusion

Research on AI tools in education has grown in recent years, especially with the development of ChatGPT, DeepSeek and similar tools. This emergence and development improved the educational system, but have created various challenges in the academic system. In particular, ethical tensions among students raised key questions in educational literature. While previous studies have examined how AI tools can affect academic integrity, critical thinking, learning growth, and academic performance among students, they have overlooked the ethical tensions embedded in different pedagogical methods in online classes. This research contributes to the literature and extends existing models of AI in education by incorporating and addressing the ethical issues and tensions perceived and experienced by teachers across five pedagogical methods in the online education system. We focused on five pedagogical methods, namely DM, CLIL, TBLT, CLT, and CLL, and interviewed 25 teachers who were engaged in online teaching. Based on the insights, we explore 20 ethical issues, each method confronting four ethical tensions. For instance, teachers experienced and perceived conversational ventriloquism, student autonomy, teacher authority, and memory erosion as key ethical tensions in DM of teaching. In the content- and language-integrated learning, teachers perceived and experienced conceptual hollowing, the bilingual bluff, the translation dependency trap, and the synthesis illusion as key ethical issues. While using a task-based learning approach, teachers experienced scaffolding collapse, the shortcut paradox, invisible inequity, and task realism corrosion as major ethical tensions. The four ethical issues, namely the authenticity crisis, risk-free fluency, the confidence mirage, and pragmatic blindness, were experienced by teachers in CLT. Finally, in the CLL approach, teachers have identified ethical issues such as the trust-erosion cycle, the AI alpha, collaborative hollowing, and the counselor’s dilemma. These ethical issues were grouped into three major domains, namely authenticity of learning, authority challenge, and collaborative dynamics, which create a causal relationship with each other. Finally, based on these ethical tensions, we developed a holistic framework that maps these ethical issues into major domains and provides a roadmap for educators and policymakers. Using this framework, policymakers can formulate strategies and tailor them to each method to improve educational quality.

## Data Availability

The original contributions presented in the study are included in the article/supplementary material, further inquiries can be directed to the corresponding author.

## Appendix

Respondent Information.

**Table 1:**

Teacher	Designation	University	Experience	Interview time (Minuts)	Interview mode
	Lecturer	Private	5 years	58	In person
	Assistant Professor	Private	8 years	55	In person
	Assistant Professor	Public	9 years	45	Online
	Assistant Professor	Private	7 years	55	Online
	Lecturer	Public	4 years	51	Online
	Associate Professor	Public	7 years	45	Online
	Associate Professor	Public	12 years	59	In person
	Assistant Professor	Public	6 years	55	Online
	Assistant Professor	Public	8 years	44	Online
	Professor	Public	19 years	55	Online
	Associate Professor	Public	13 years	45	Online
	Lecturer	Public	3 years	56	Online
	Assistant Professor	Public	8 years	47	Online
	Assistant Professor	Private	8 years	56	Online
	Professor	Public	16 years	48	Online
	Associate Professor	Public	12 years	49	In person
	Associate Professor	Public	11 years	46	In person
	Lecturer	Public	4 years	51	In person
	Lecturer	Private	4 years	58	Online
	Professor	Public	19 years	47	Online
	Professor	Public	18 years	45	Online
	Assistant Professor	Public	8 years	65	In person
	Lecturer	Public	4 years	48	Online
	Lecturer	Public	8 years	45	Online
	Lecturer	Private	5 years	50	In person
